# A retrospective single-center pilot study of the genetic background of the transplanted kidney

**DOI:** 10.1371/journal.pone.0316192

**Published:** 2025-01-08

**Authors:** Anna Novotna, Klara Horackova, Jana Soukupova, Petra Zemankova, Petr Nehasil, Pavel Just, Ludek Voska, Petra Kleiblova, Silvie Rajnochova Bloudickova

**Affiliations:** 1 Department of Nephrology, Institute for Clinical and Experimental Medicine, Prague, Czech Republic; 2 First Faculty of Medicine, Institute of Medical Biochemistry and Laboratory Diagnostics, Charles University and General University Hospital in Prague, Prague, Czech Republic; 3 First Faculty of Medicine, Institute of Pathological Physiology, Charles University, Prague, Czech Republic; 4 Department of Pediatrics and Inherited Metabolic Disorders, First Faculty of Medicine, Charles University and General University Hospital in Prague, Prague, Czech Republic; 5 Department of Clinical and Transplant Pathology, Institute for Clinical and Experimental Medicine, Prague, Czech Republic; 6 First Faculty of Medicine, Institute of Biology and Medical Genetics, Charles University and General University Hospital in Prague, Prague, Czech Republic; Memorial Sloan Kettering Cancer Center, UNITED STATES OF AMERICA

## Abstract

**Introduction:**

Renal cell carcinoma (RCC) is one of the most prevalent cancers in kidney transplant recipients (KTR). The hereditary background of RCC in native kidneys has been determined, implicating its clinical importance.

**Materials and methods:**

This retrospective single-center pilot study aimed to identify a potential genetic predisposition to RCC of the transplanted kidney and outcome in KTR who underwent single kidney transplantation between January 2000 and December 2020 and manifested RCC of the transplanted kidney. Next-generation sequencing (NGS) based germline genetic analysis from peripheral blood-derived genomic DNA (gDNA) was performed in both the recipient and donor using a gene panel targeting 226 cancer predisposition genes.

**Results:**

The calculated incidence of RCC of the transplanted kidney among 4146 KTR was 0.43%. In fifteen KTR and donors, NGS was performed. The mean KTR age at transplantation and the diagnosis of RCC was 50.3 years (median 54; 5–67 years) and 66 years (median 66; 24–79 years), respectively. The mean donor age at transplantation and graft age at RCC diagnosis was 39.7 years (median 42; 7–68 years) and 50.2 years (median 46; 20–83 years), respectively. The mean follow-up after RCC diagnosis was 47 months (median 39.1; 0–112 months). Papillary RCC was the most prevalent (n = 8), followed by clear cell RCC (n = 6) and unspecified RCC (n = 1). Thirteen RCCs were low-stage (pT1a/b) diseases, one was pT3, and one was of unknown stage. Most RCC was higher graded. No germline pathogenic cancer-predisposition variant was found in either KTR or donors except for several variants of uncertain significance.

**Conclusion:**

RCC of the transplanted kidney is very rare. Germline cancer-predisposition testing has identified several variants of uncertain significance, but no germline genetic predisposition to graft RCC in KTR. Further research is needed to assess the clinical relevance of genetic testing for cancer risk in KTR.

## Background

Cancer is the second leading cause of mortality in kidney transplant recipients (KTR) with previously reported overall 2 to 3-fold increased risk of cancer and cancer-related death in KTR compared to age- and gender-matched general population [1–3]. The increased cancer risk can be pinned on several factors related to immunity, genetics, and environmental exposures. Specifically, long-term immunosuppressive medication and chronic antigen stimulation from the graft accompanied by decreased immunosurveillance, exposure to oncogenic viruses and ultraviolet (UV) light, and inherited genetic cancer predispositions, may contribute to kidney tumor formation [4].

Renal cell carcinoma (RCC) is one of the most prevalent cancer types in KTR. The risk of RCC has been reported to be 5 to 10 times higher than that of the general population [5]. Common RCC risk factors in KTR are older age, male sex, obesity, smoking, and acquired cystic kidney disease (ACKD) [6–8]. Similarly, risk factors of the donors include older age, hereditary renal disease, or cancer history [9, 10].

Most RCC occurs in the native kidney as opposed to the transplanted kidney [11–13]. Reported RCC incidence is about 1.55% compared to the incidence in transplanted kidneys varying between 0.2% and 0.5% [5, 14, 15]. There are two pathways of kidney graft cancer formation—either *de novo* development of the KTR origin or derived from a donor. A transmission of microscopic RCC to the recipient via the allograft has been well documented [16].

Recently, it has been estimated that 2–8% of RCC cases are of hereditary origin with identified germline pathogenic or likely pathogenic variants (GPV) in genes including *BAP1*, *FH*, *FLCN*, *MET*, *MITF*, *PTEN*, *SDH* genes (*SDHA*, *SDHAF2*, *SDHB*, *SDHC*, *SDHD)*, *TSC1*, *TSC2*, *VHL*, and *WT1* [17–22]. Moreover, RCC patients reportedly harbored GPV in high-to-moderate penetrance genes associated with other hereditary cancer syndromes, including *APC*, *ATM*, *BARD1*, *BRCA1*, *BRCA2*, *CHEK2*, *MLH1*, *MSH2*, *MSH6*, and *PMS2* [22, 23]. The guidelines for germline genetic testing for hereditary renal cell carcinoma syndromes have been established recently [24].

We hypothesize that the risk of *de novo* cancer of the transplanted kidney might be associated with carriership of GPV in a cancer-predisposition gene (CPG) of either the donor or the recipient, as has been previously reported in cases of other tumors developed after kidney transplantation [25, 26]. In this pilot study, we aimed to analyze the GPV in established, as well as potentially candidate RCC predisposition genes in a retrospective, single-center cohort of KTR with manifested cancer of the transplanted kidney. Identification of carriership of GPV in a cancer-predisposition gene may be useful to establish an individualized oncological screening, which would contribute to early detection of possible cancer and thus reduce post-transplant malignancy-associated morbidity and mortality.

## Materials and methods

### 1. Patients and controls

This retrospective single-center pilot study was conducted with patients who underwent kidney transplantation at the Institute for Clinical and Experimental Medicine (IKEM). Criteria for inclusion in the study were a history of kidney transplantation, a history of cancer of the transplanted kidney in KTR, the availability of DNA, and of kidney recipient and donor-relevant clinical data. Clinical data of the kidney recipient and his donor are stored in the hospital database in electronic and paper form as standard. These data are entered at the time of acceptance of the kidney donor to the kidney recipient from the waiting list of the transplantation center according to the allocation rules of the Czech Republic. KTR are further monitored as a part of regular check-ups at IKEM.

**Fig 1:**
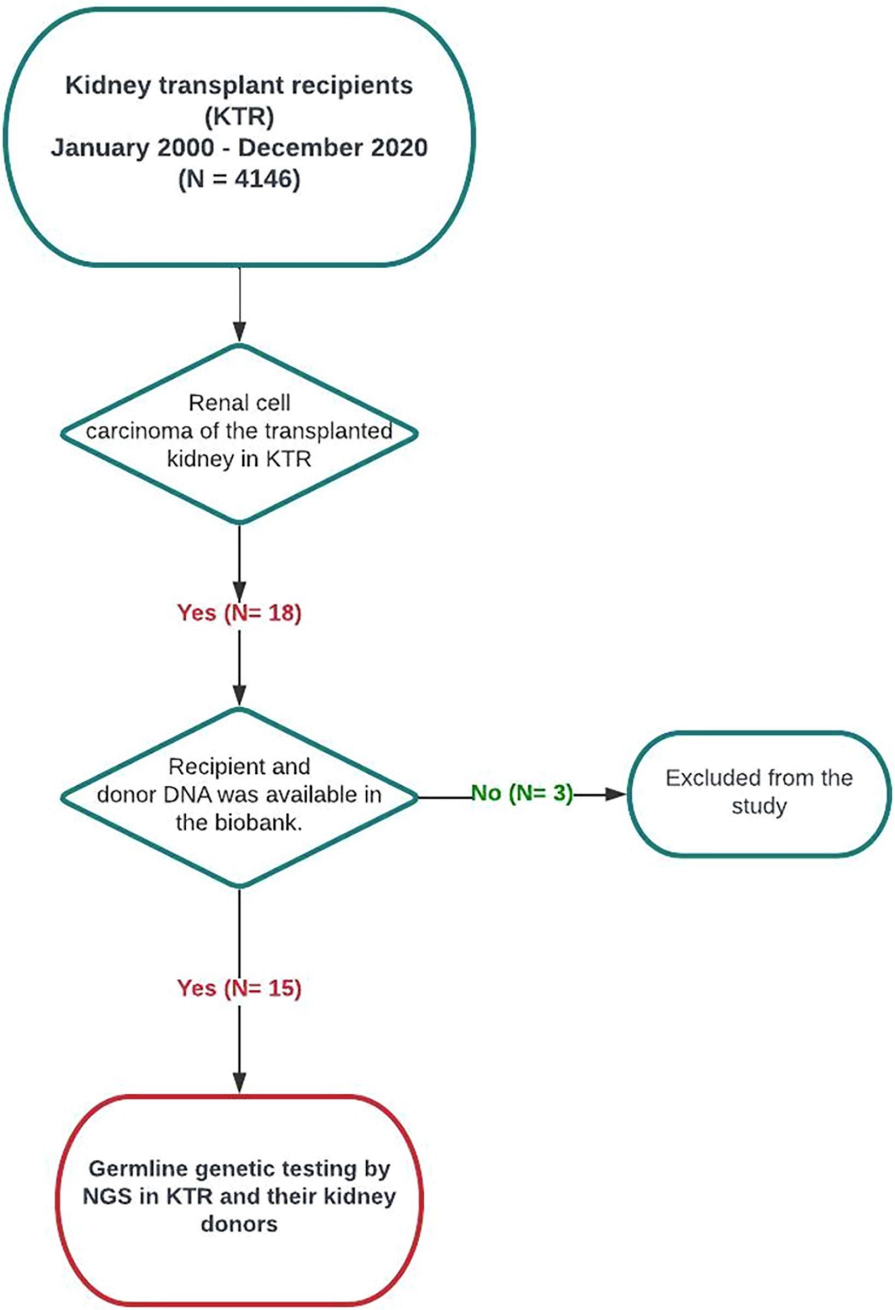
Study flowchart. NGS, next-generation sequencing.

Out of 4146 Caucasian patients who have been transplanted between January 2000 to December 2020 at IKEM, eighteen KTR have been identified to develop RCC of the transplanted kidney, nine females and nine males. The study subgroup for germline genetic testing finally consisted of 15 KTR and their graft donors whose DNA we had for disposal (Fig 1).

During November 1–30, 2023 demographic, clinical, and histopathological data were extracted from the hospital information system of IKEM. Following the local legislation and institutional requirements, the patients gave written informed consent to storing their blood samples, agreed to use their blood samples, including genetic testing, and to use their medical records for future research, approved by the Ethics Committee of the Institute for Clinical and Experimental Medicine and Thomayer Hospital (Ethic Board Approval A13-02-01 for biobanking).

Two groups of controls were used for the genetic case-control study: i) “super-controls” (healthy individuals ≥60 years with no personal and family cancer history; N = 616) were used for variant prioritization; and ii) previously described population-matched controls provided by the National Center for Medical Genomics (N = 1 662; http://ncmg.cz; [27]) for statistical case-control analysis.

### 2. Methods

Germline genetic testing of the KTR and their donors was performed by next-generation sequencing (NGS) using the custom CZECANCA panel (CZEch CAncer paNel for Clinical Application; Roche, Basel, Switzerland) targeting 226 cancer predisposition genes, as described previously with minor modifications (28,29). Briefly, the NGS pre-library was prepared using the KAPA EvoPlus Kit (Roche, Basel, Switzerland) with the input of 200 ng peripheral blood-derived genomic DNA (gDNA) according to the manufacturers’ instructions using in-house designed adapters, dual indexes, and primers. The final library was sequenced using the NextSeq 500/550 Mid Output Kit v2.5 (150 cycles) on an Illumina NextSeq 500 instrument (Illumina, San Diego, CA, USA). Subsequent bioinformatic analysis of the NGS data was performed as described previously and included single nucleotide polymorphism (SNP), small insertions/deletions (indels), as well as copy number variation (CNV) analysis [28, 29].

The identified GPVs were prioritized as described previously with minor modifications [29]. Briefly, we removed variants with insufficient sequencing quality; located in repetitive, low-complexity, and non-coding regions; present in super-controls and population databases with a frequency >0.004; classified in ClinVar as benign/likely benign; intronic variants outside the canonical splice-sites (+/-1,2 bp), and synonymous or missense variants unless leading to aberrant splicing or classified pathogenic/likely pathogenic in ClinVar with at least two submitters with no conflict.

The prioritized GPV in 226 tested genes were divided into 4 groups, according to whether they have been associated with predisposition to: RCC (group 1), hereditary cancer syndromes that also manifest with RCC (group 2), other hereditary cancer syndromes (group 3), and other not yet clinically relevant cancer predisposition genes (group 4; data in S1 Table). The GPV in clinically relevant cancer predisposition genes (groups 1–3) was confirmed by Sanger sequencing or MLPA (primers available upon request).

The statistical analysis of the genetic case-control study was performed in R version 4.3.2. Pairwise comparison was performed using Fisher’s exact test; p <0.05 was considered significant.

## Results

### 1. Clinicopathological characteristics and associations

The calculated incidence of RCC of the transplanted kidney was 0.43%. The average KTR age at transplantation and the diagnosis of RCC of the transplanted kidney was 50.3 years (median 54; 5–67 years) and 66 years (median 66; 24–79 years), respectively. The etiology of end-stage kidney disease (ESKD) was polycystic kidney disease (n = 4), membranoproliferative glomerulonephritis (n = 2), anti-GBM glomerulonephritis (n = 1), Alport syndrome (n = 1), congenital disorder (n = 1), hypertensive nephropathy (n = 1), renal amyloidosis (n = 1), and unknown (n = 4). The average dialysis vintage was 25 months (median 19; 3–77 months). The majority of KTR were non-smokers without cardiovascular disease or diabetes. None of the KTRs had a history of cancer before transplantation. All KTR had calcineurin-based immunosuppression at the time of RCC diagnosis, in 13 in combination with an antiproliferative agent. The mean donor age at transplantation was 39.7 years (median 42; 7–68 years) and the graft age at RCC diagnosis was 50.2 years (median 46; 20–83 years). All donors were unrelated to their recipient (Table 1).

**Table 1:** Patient characteristics.

Patient No.	Sex (M/F)	Dialysis vintage (months)	Cause of ESKD	Hypertension	Cardiovascular disease	Smoking	Cancer prior transplantation	Diabetes mellitus	BMI (kg/m2)	Year of transplantation	Age at transplantation (years)	Age at RCC diagnosis (years)	Donor age (years)	Graft age at RCC diagnosis (years)	Graft function at RCC diagnosis (ml/s/1.73m2)	IS at RCC diagnosis
1	M	31	Unknown	Yes	Yes	No	No	No	26.2	2007	30	36	48	55	0.83	TAC, MMF, KS
2	M	26	Unknown	Yes	No	No	No	No	26.9	2003	59	69	46	56	0.79	CyA, MMF, KS
3	M	19	PKD	Yes	Yes	No	No	No	24.7	1999	64	79	30	46	0.51	CyA, KS
4	F	31	Anti-GBM GN	Yes	No	Yes	No	No	32.7	2008	54	61	38	45	0.48	CyA, MMF, KS
5	M	23	MPGN	Yes	No	No	No	No	25.5	2004	53	66	7	20	1.53	CyA, MMF, KS
6	F	11	Alport syndrome	Yes	No	No	No	No	18	2001	33	49	68	83	0.27	TAC, MMF, KS
7	M	14	PKD	Yes	Yes	Yes	No	No	20.2	2015	67	68	62	64	0.49	TAC, MMF, KS
8	F	78	Unknown	Yes	No	No	No	No	22.8	2003	43	56	17	30	0.53	TAC, MMF, KS
9	F	3	MPGN	Yes	No	Yes	No	No	21	2014	63	67	57	61	0.49	TAC, MMF, KS
10	F	6	PKD	Yes	No	Yes	No	No	23.9	2005	50	63	56	69	1.15	TAC, MMF, KS
11	F	77	Amyloidosis	Yes	No	No	No	No	22.8	2003	50	69	51	69	0.27	TAC, KS
12	M	22	Hypertension	Yes	No	No	No	No	29.9	2020	64	65	42	43	1.41	TAC, MMF, KS
13	F	13	Anti-GBM GN	Yes	No	Yes	No	No	22.7	2006	54	68	26	41	0.93	TAC, MMF, KS
14	M	8	PKD	Yes	No	No	No	No	25.7	2016	66	71	36	41	0.8	TAC, MMF, KS
15	M	13	CAKUT	Yes	No	Yes	No	No	30.3	2000	5	24	12	30	2.01	TAC, MMF

AZA, azathioprine; BMI, body mass index; CAKUT, congenital anomalies of the kidney and urinary tract; CS, corticosteroids; CyA, cyclosporine; ESKD, end stage kidney disease; GBM, glomerular basal membrane; GN, glomerulonephritis; MMF, mycophenolate mofetil; MPGN, membranoproliferative glomerulonephritis; PKD, polycystic kidney disease; RCC, renal cell carcinoma; TAC, tacrolimus.

The graft function at RCC diagnosis varied between 0.27 to 1.41 ml/s/1.73m^2^ (median 0.79). The average time between transplantation and RCC diagnosis was 124.3 months (median 147; 17–214 months). The mean follow-up after RCC diagnosis was 47 months (median 39.1; 0–112 months) (Table 2).

**Table 2:** Characteristics of transplanted kidney RCC.

Patient No.	Time between transplantation and diagnosis (months)	Type of RCC (histology)	Stage (AJCC)	Grade	Therapy	RCC outcome. (solved, relapsed)	Graft outcome (funtion/afunction)	Patient outcome (alive, death)	Follow-up after RCC diagnosis (months)
1	76	Papillary	pT1a pN0 pMX, stage I	G2	Resection	Solved	Function	Alive	112
2	122	Clear cell	pT1a pNX pMX, stage I	G3	Resection	Relapsed	Function	Alive	112
3	186	Papillary	pT1a pNX pMX, stage I	G1	Resection	Solved	Function	Death	17
4	87	Papillary	pT1 pN0 pMX, stage I	G3	Resection	Solved	Function	Alive	87
5	147	Papillary	pT1a pN0 pM x, stage I	G1	Resection	Solved	Function	Death	55
6	185	Clear cell	pTa pNX pMX, stage I	G2	Resection	Solved	Afunction	Alive	13
7	17	Papillary	pT1a pNX pMX, stage I	G1	Resection	Solved	Function	Death	17
8	161	Papillary	pT1a pNX pMX, stage I	G3	Resection	Solved	Function	Alive	72
9	41	Clear cell	pT1b pNX pMX, stage I	G2	Resection	Solved	Function	Alive	29
10	154	Papillary	pT1a pNX pMX, stage I	G1-2	Resection	Relapsed	Function	Alive	61
11	226	Unspecified	pT3 pNX pMX, stage III	G3	Graftectomy	Solved	Graftectomy	Death	0
12	15	Clear cell	pT1a pNX pMX, stage I	G1	Resection	Solved	Function	Death	11
13	173	Papillary	pT1a pNX pMX, stage I	G1-2	Resection	Solved	Function	Alive	31
14	61	Clear cell	Unknown	G2	Resection	Solved	Function	Alive	28
15	214	Clear cell	pT1a pNX pMX, stage I	G1	Resection	Solved	Function	Alive	63

AJCC, American Joint Commitee on Cancer; RCC, renal cell carcinoma.

### 2. RCC of the transplanted kidney

The most prevalent was papillary RCC (n = 8) followed by clear cell RCC (n = 6) and unspecified RCC (n = 1). Most KTR had low-stage (pT1a/b) disease (n = 13), one patient had pT3 and one patient had an unknown stage. The majority of RCC was higher graded (Table 2). All RCCs of the transplanted kidney except for one were treated with surgical resection. One patient underwent graftectomy because of adverse tumor location and very limited function of the transplanted kidney. Two patients underwent successful RCC re-resection because of relapse. Only one patient lost his function of the transplanted kidney after RCC resection but after ten years. Five patients died an average of 20 months after RCC diagnosis (median 17; 0–55 months). Ten patients stayed alive with a mean follow-up of 60.8 months (median 61; 13–112 months).

### 3. Genetic analysis

We analyzed germline variants (SNP, indels, and CNV) in 15 KTR and their 15 kidney donors using the CZECANCA NGS panel targeting 226 CPG (data in S1 Table). A total of 65 variants (data in S2 Table) remained for further evaluation after prioritization. However, we identified no germline pathogenic variant (GPV) in renal cancer predisposition genes (*BAP1*, *FH*, *FLCN*, *MET*, *PTEN*, *SDHA*, *SDHAF2*, *SDHB*, *SDHC*, *SDHD*, *TSC1*, *TSC2*, *VHL*, WT1) and other clinically relevant CPG (group 2 and 3, data S1 Table) in either KTR or a kidney graft donor. However, we found 10 variants of uncertain significance (VUS) in clinically relevant CPG (groups 1–3) in 5 KTR and 3 donors (one KTR and one donor carried VUS in two genes, Table 3), including 1×VUS in the *SDHB* gene encoding the succinate dehydrogenase (SDH) complex subunit associated with kidney cancer risk. In addition, one KTR harbored a GPV in another tested gene from group 4, a drug-toxicity associated gene *DPYD* (Dihydropyrimidine Dehydrogenase), and one donor harbored a GPV in *MPL* (Myeloproliferative Leukemia Oncogene; Table 3).

**Table 3:** Identified variants in KTR and their donors using the CZECANCA panel. All variants were present in heterozygous state.

Patient No.	Kidney transplant recipient		Kidney donor	
	VUS in established CPG*	GPV in other genes	VUS in established CPG*	GPV in other genes
1	*SDHB*:c.298T>A/p.Ser100Thr; *POLE*:c.2683G>A/p.Ala895Thr	-	-	-
2	-	-	-	-
3	*BRCA2*:c.8393C>G/p.Pro2798Arg	-	*MSH2*:c.403C>G/p.Leu135Val; *RAD51D*:c.412A>G/p.Asn138Asp	-
4	-	-	-	-
5	*STK11*:c.841C>A/p.Pro281Thr	-	*MSH6*:c.3961A>G/p.Arg1321Gly	-
6	*ATM*:c.1751A>G/p.Gln584Arg	-	-	-
7	-	-	-	-
8	-	-	-	-
9	-	-	-	-
10	*APC*:c.5362C>A/p.Arg1788Ser	-	*ATM*:c.6844A>G/p.Asn2282Asp	-
11	-	-	-	-
12	-	*DPYD*:c.1905+1G>A	-	-
13	-	-	-	*MPL*:c.1653+1delG
14	-	-	-	-
15	-	-	-	-

* List of established high-to-moderate penetrance cancer-predisposition genes (group 1–3) is available in data S1 Table. A complete list of identified variants left after filtration and prioritization is available in data S2 Table.

VUS, variant of uncertain significance; CPG, cancer-predisposition gene; GPV, germline pathogenic/likely-pathogenic variant.

Interestingly, we identified a total of three variants (yet of uncertain significance) in *NFKBIZ* (Nuclear Factor Kappa-B Inhibitor Zeta)—twice the same missense variant c.1061C>G (p.Ala354Gly) in two donors and once the missense variant c.1661C>T (p.Thr554Ile) in one patient. The variant *NFKBIZ*:c.1061C>G identified in two donors is currently classified in a ClinVar as VUS by single submitter due to its rarity in population databases (maximum allelic frequency in genomAD 0.0003) and insufficient knowledge of its functional impact. The c.1061C>G variant is present only twice in our population-matched controls and is therefore significantly enriched in the case-control analysis in the group of kidney transplant donors (2/1662, 0.001; vs. 2/15 donors, 0.1; *p* = 4˟10^−4^).

## Discussion

Cancer with cardiovascular and infectious complications belongs to the main causes of death in KTR (1,2,3). Post-transplant, non-melanoma skin cancer (NMSC) predominates as the most frequent cancer followed by post-transplant lymphoproliferative disorder (PTLD), gastrointestinal and genitourinary cancers, of which RCC is the most common urological cancer in KTR with the incidence varying from 0.25 to 5.23% [30, 31]. RCC can affect native kidneys or rarely transplanted kidneys, however the mechanism is poorly understood.

In our large cohort of 4146 KTR, cancer of the transplanted kidney develops in only 0.43% of them which is consistent with the previous studies describing the incidence as around 0.2% [32–34]. The median time to RCC of the transplanted kidney diagnosis was 147 months (12.25 years), with the median age of KTR and of the graft at the RCC diagnosis being 66 and 46 years, respectively. There is only limited recent data concerning the period between transplant surgery and the onset of RCC in KTR. Reported findings widely range from 5 to 452 months, most often within 6–7 years post-transplant [35, 36]. Similar to our observation, in a French multicentric study Tillou et al. reported the mean time to cancer of the transplanted kidney diagnosis 132 months (11 years) with a patient’s mean age of 47 years [37]. In a single-center retrospective study, Leveridge et al. showed the mean time to cancer of the transplanted kidney diagnosis 12.1 years post-transplant with papillary and clear cell RCC as represented histological subtypes of RCC [11]. We also predominantly found papillary cell and clear cell RCC, in a similar rate of 53.3%, resp. 40% of KTR. Being the retrospective but largest study so far, Szabla et al. recently reported that RCC of the transplanted kidney seems to be a different entity with a higher incidence of papillary cell RCC and of lower stage [38]. In contrast, the most prevalent RCC in native kidneys is clear cell RCC (70–80%), papillary cell RCC (10–15%), and chromophobe RCC (5%).

RCC was considered to be a predominantly sporadic cancer (about 90–95%) but increasing evidence of the genetic link between sporadic and syndromic RCC is becoming more evident [39]. Risk factors for RCC in KTR include older age, male sex, longer dialysis vintage (≥3 years), and ESKD due to glomerular disease, hypertensive or vascular nephropathy [40]. In our cohort, apart from older age (median 66 years) and hypertension present in all KTR, we haven’t found other common risk factors mentioned in the literature. Except for two patients, all were managed with the same concomitant immunosuppressive therapy based on calcineurin inhibitor (CNI) and mycophenolate mofetil (MMF). Thus, it did not seem to have a specific effect on the development of RCC of the transplanted kidney. Current data haven’t yet supported the hypothesis that any particular drug combination is more harmful [41, 42].

Post-transplant compromised immunity is proposed to be a major contributor to increased cancer risk leading to *de novo* sporadic tumor formation, but hereditary cancer predisposition coming either from the KTR or the donor may also be involved [3]. The cases of donor-transmitted or -derived cancer, including hematological, skin, urothelial, and neuroendocrine malignancies, in transplant recipients are rare and have been previously well documented [25, 26, 43–46]. Some authors have considered cancer manifestation less than 2 years after transplantation as transmission from the donor [47, 48]. However, as only short tandem repeats (STR) genotyping of the donor/recipient cell origin is commonly performed, we lack information on the genetic cancer predisposition of the donor or the recipient.

We performed a wider germline genetic testing using panel NGS of 226 CPG in 15 KTR and their unrelated donors; however, we failed to identify GPV in CPG as we originally presumed, which might be caused by the limited size of our study group.

Despite limited evidence of graft-derived tumors in KTR, a few cases have been published. Specifically, one case of thoroughly characterized KTR with donor-derived skin squamous cell carcinoma with identified GPV in *TP53* has been reported [43]. *TP53* is an established, high-penetrance cancer-predisposition gene causing Li-Fraumeni syndrome typical of sarcomas, brain tumors, breast, and adrenocortical carcinomas, but sometimes also manifesting with skin cancer which is the most common cancer in KTR [49]. Similarly, a case report about a follicular lymphoma found in both the KTR and his daughter (the kidney donor) has been published describing a shared mutation pattern suggesting both lymphomas originated from one donor-derived precursor [44]. These results point towards the genetic cancer predisposition provided by the donor that contributed to the tumor development in KTR, analogically to our experiment hypothesis.

Besides the lack of GPV in CPG, we identified ten VUS in established high-to-moderate clinically relevant CPG. Furthermore, we identified the c.1061C>G VUS in *NFKBIZ* identified in two donors. Interestingly, dysregulated NFKBIZ (sometimes also called IKBZ or INAP) signaling is associated with acute kidney injury and associated immune response [50–52]. More specifically, it has been suggested that NFKBIZ is involved in renal fibrosis and, analogically to liver fibrosis leading to hepatocellular carcinoma, we can speculate that NFKBIZ-mediated fibrosis in donor’s kidney may lead to tumor of the transplanted kidney in the recipient, especially when combined with the recipients’ compromised immunosurveillance due to long-term immunosuppressive treatment [53–55]. Nevertheless, without further research including extensive case-control studies analyzing the variant frequency in kidney donors and KTR with cancer of the transplanted kidney, as well as functional studies of the variant impact, no conclusion about c.1061C>G correlation with cancer of the transplanted kidney can be up-to-date drawn. In general, VUS represents a major group of variants identified by NGS, and their further reclassification–either to benign/likely benign or pathogenic/likely pathogenic—is the main challenge of current variant classification. Accordingly, the identification of not only GPV but also VUS in patients with suspected genetic cancer predisposition is critical for variant reclassification and thus potentially for patients’ management [56–59].

Similar to our study on KTR, a germline genetic testing of recipients of allogeneic hematopoietic stem cell transplantation (HSCT) was performed aiming to identify GPV in clinically relevant–disease-predisposing, or therapy-related—genes to hematological, as well as solid malignancies [60]. In the Finish study by Lahtinen *et al*. [60], a few carriers of high-penetrance cancer predisposition genes were identified, including *BRCA1*, *BRCA2*, and *PALB2* (each once in adult patients with hematological malignancies). Interestingly, 37% of adult recipients from related donors were carriers of GPV emphasizing the importance of the germline genetic testing of recipients, as well as the related donors before transplant.

Similar conclusions stressing out the need for pre-transplant germline genetic testing may be proposed for other types of malignancies with established genetic predisposition, including RCC, which could improve the long-term survival of patients including prevention of post-transplant malignancies, therapy efficacy, or drug toxicity.

The implementation of germline genetic testing in patients with end-stage kidney failure due to potentially inherited kidney disease is currently under discussion when considering patient-related living kidney donors who may carry the same inherited GPV [61]. It has also been documented that kidneys transplanted from older donors have a higher risk of developing graft tumors. Therefore, it can be hypothesized that older donor-derived tumors are more likely to be sporadic, but when using a kidney from a younger donor, the potential risk of hereditary cancer is higher. Thus, germline genetic testing for cancer-predisposition GPV in younger donors may be even more beneficial for cancer-focused post-transplant care in KTR [9, 10, 62]. Pre-transplant knowledge of the carriership of hereditary cancer predisposition genes in KTR could be beneficial for the implementation of individualized oncological screening for early detection of possible post-transplant cancer, as well as for the assessment of possible risks of potential living related donor as a part of the framework of a comprehensive examination before kidney donation. However, as our study group did not include any donors related to their recipients, we cannot draw now any specific conclusions regarding efficacy of donor-recipient germline genetic testing from our data.

Currently, the guidelines for clinical practice for carriers of pathogenic variants in clinically relevant cancer predisposition genes are already established and are based on the current recommendations of the National Comprehensive Cancer Network (NCCN) and European Society of Medical Oncology (ESMO) [63, 64]. These guidelines define the primary and secondary prevention, especially in previously oncologically healthy individuals at high risk of cancer caused by germline mutations in the highly and moderately penetrant cancer predisposition genes BRCA1, BRCA2, PALB2, ATM a CHEK2, which occur most frequently in breast, and ovarian cancers, prostate, pancreas, but also other organs [65]. Pre-transplant germline genetic testing might contribute to the reduction of cancer related mortality, the second cause of death in kidney transplant patients as well as the general population in the Czech Republic [66].

Limitations of our study include the small size of the donor/recipient study group due to the low prevalence of kidney graft RCC; however, to the best of our knowledge, it is the largest genetically analyzed group to date. Moreover, using the CZECANCA NGS panel (data in S1 Table), we mainly tested genes established in predisposition to solid tumors including some, but not all genes known to predispose to RCC such as *MITF*, which was not included in our panel, but its GPV have been reported in patients with melanoma and RCC [67]. In addition, chromosomal aberrations were not assessed, thus leaving a gap for example in hereditary RCC syndrome caused by chromosome 3 translocation [68].

## Conclusion

In our pilot study analyzing 15 pairs of kidney transplant recipients who developed graft tumor and their donors, we identified no germline pathogenic variant suggesting the involvement of an established genetic cancer predisposition in either recipients or donors. In addition, we identified several variants of uncertain significance that require further investigation to establish their pathogenicity and transplanted kidney tumor association. Nevertheless, KTR (and their donors) may benefit from germline genetic testing of cancer predisposition as prevention of malignancy, as well as when considering a related living donor.

## Supporting information

S1 Table List of 226 cancer-predisposition genes included in CZECANCA panel. (XLSX)

S2 Table Identified variants in 15 kidney transplant recipients and donors. (XLSX)
